# Marked Response to Immunochemotherapy in Airway Mucosal Recurrence of Lung Squamous Cell Carcinoma

**DOI:** 10.1093/icvts/ivag193

**Published:** 2026-07-06

**Authors:** Zijian Li, Chudong Wang, YenZhir Tay, Shuben Li

**Affiliations:** Department of Thoracic Surgery and Oncology, the First Affiliated Hospital of Guangzhou Medical University, State Key Laboratory of Respiratory Disease & National Clinical Research Center for Respiratory Disease, Guangzhou 510120, China; Department of Thoracic Surgery and Oncology, the First Affiliated Hospital of Guangzhou Medical University, State Key Laboratory of Respiratory Disease & National Clinical Research Center for Respiratory Disease, Guangzhou 510120, China; Department of General Surgery, Thoracic Unit, Kuala Lumpur Hospital (HKL), Kuala Lumpur, 50586, Malaysia; Department of Thoracic Surgery and Oncology, the First Affiliated Hospital of Guangzhou Medical University, State Key Laboratory of Respiratory Disease & National Clinical Research Center for Respiratory Disease, Guangzhou 510120, China

**Keywords:** immunochemotherapy, airway mucosal recurrence, lung squamous cell carcinoma, airway recurrence

## Abstract

**Objectives:** Airway mucosal recurrence after complete resection of primary lung cancer is rare and lacks standard treatment. We aim to report a marked response to immunochemotherapy in a patient with multifocal airway mucosal recurrence of lung squamous cell carcinoma.

**Case description:** A 50-year-old male patient presented with an irritative cough 2 years after right upper sleeve lobectomy for lung squamous cell carcinoma (SCC) (pT1bN0M0). Computed tomography and bronchoscopy revealed multiple recurrent polypoidal lesions in the trachea, with biopsy confirming recurrent SCC. After restaging and multidisciplinary evaluation, the patient was offered immunochemotherapy. The recurrent mucosal lesions exhibited a favourable response to the combination of a PD-1 inhibitor and chemotherapy.

**Conclusions:** This case suggests a potentially effective strategy for high-risk patients with airway mucosal recurrence of lung squamous cell carcinoma.

## INTRODUCTION

Airway mucosal recurrence after complete resection of a primary lung cancer is uncommon, with a reported prevalence of 0.44%.^1^ It is typically associated with bronchioloalveolar carcinoma, whereas other types of non-small cell lung cancer (NSCLC) rarely develop intra-airway dissemination. Currently, there is a lack of consensus on effective treatment options for airway mucosal recurrence. Immune checkpoint inhibitors are a promising treatment for NSCLC, especially for lung squamous cell carcinoma (SCC). Combination therapies of immunochemotherapy or immunotherapy with radiotherapy in NSCLC demonstrate a significant improvement in clinical prognosis and survival outcomes. However, the efficacy of immunochemotherapy for multifocal airway mucosal recurrence remains relatively unknown.

## CASE REPORT

**Figure 1.**
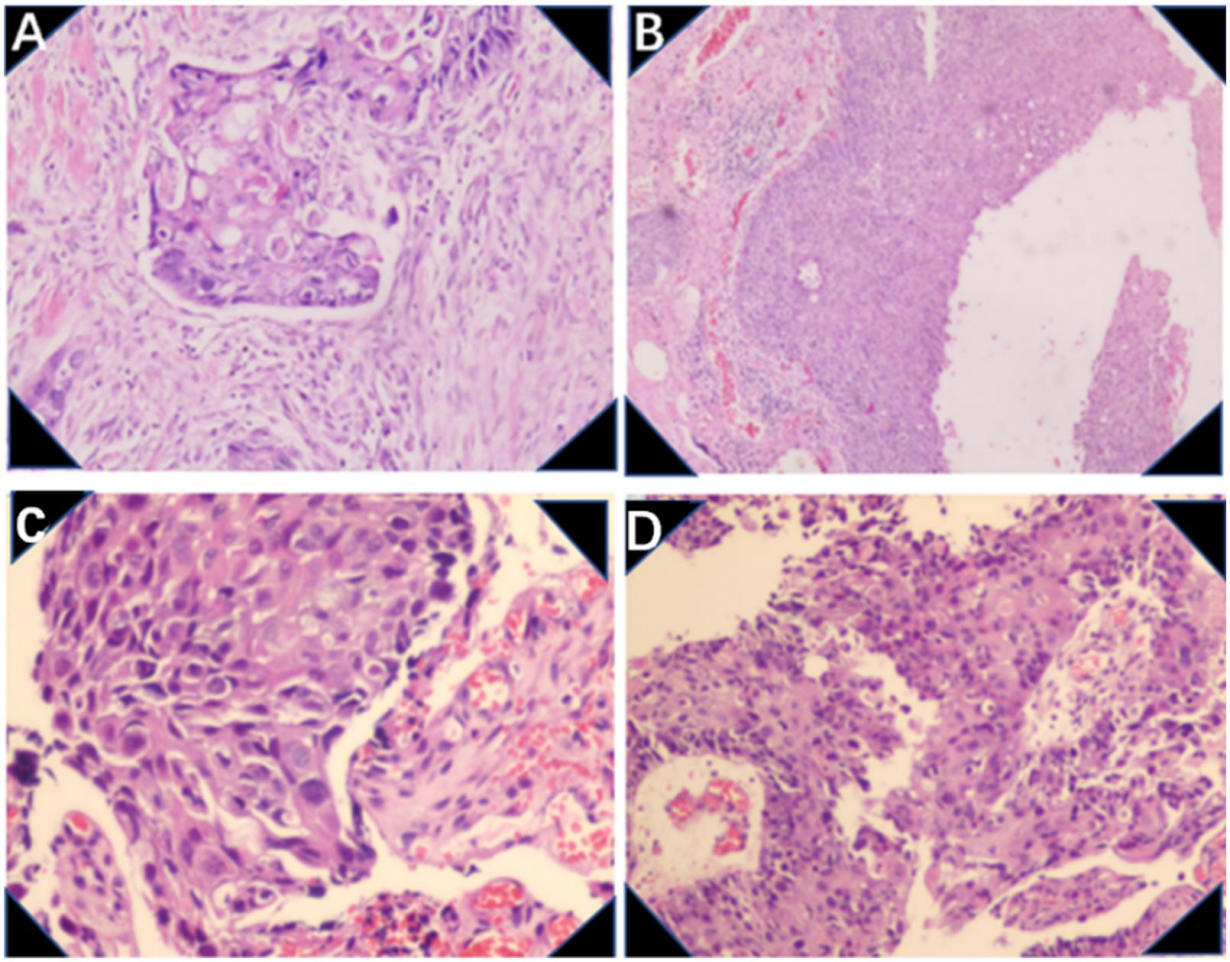
(A and B) Postoperative Pathology Indicated Keratinizing Squamous Cell Carcinoma with Moderate Differentiation. Invasion of the right upper lobar bronchial wall and nerve bundle could be observed. (Hematoxylin and Eosin [H&E] Stain, X100). (C and D) The H&E Pathological Specimens of the Polypoidal Lesions in the Tracheal Wall, Squamous Cell Carcinoma Component. (Hematoxylin and Eosin [H&E] Stain, X100).

A 50-year-old man underwent right upper sleeve lobectomy with radical lymphadenectomy for lung cancer, and he recovered well without postoperative complications. The tumour was strictly confined to the right upper lobar bronchus orifice without main bronchus involvement. Postoperative pathology confirmed cartilaginous layer invasion without breaching the adventitia, negative margins (>0.5 cm), and negative nodes (0/37). The staging was confirmed as pT1bN0M0 (stage IA2) (**Figure 1A and B**).

**Figure 2.**
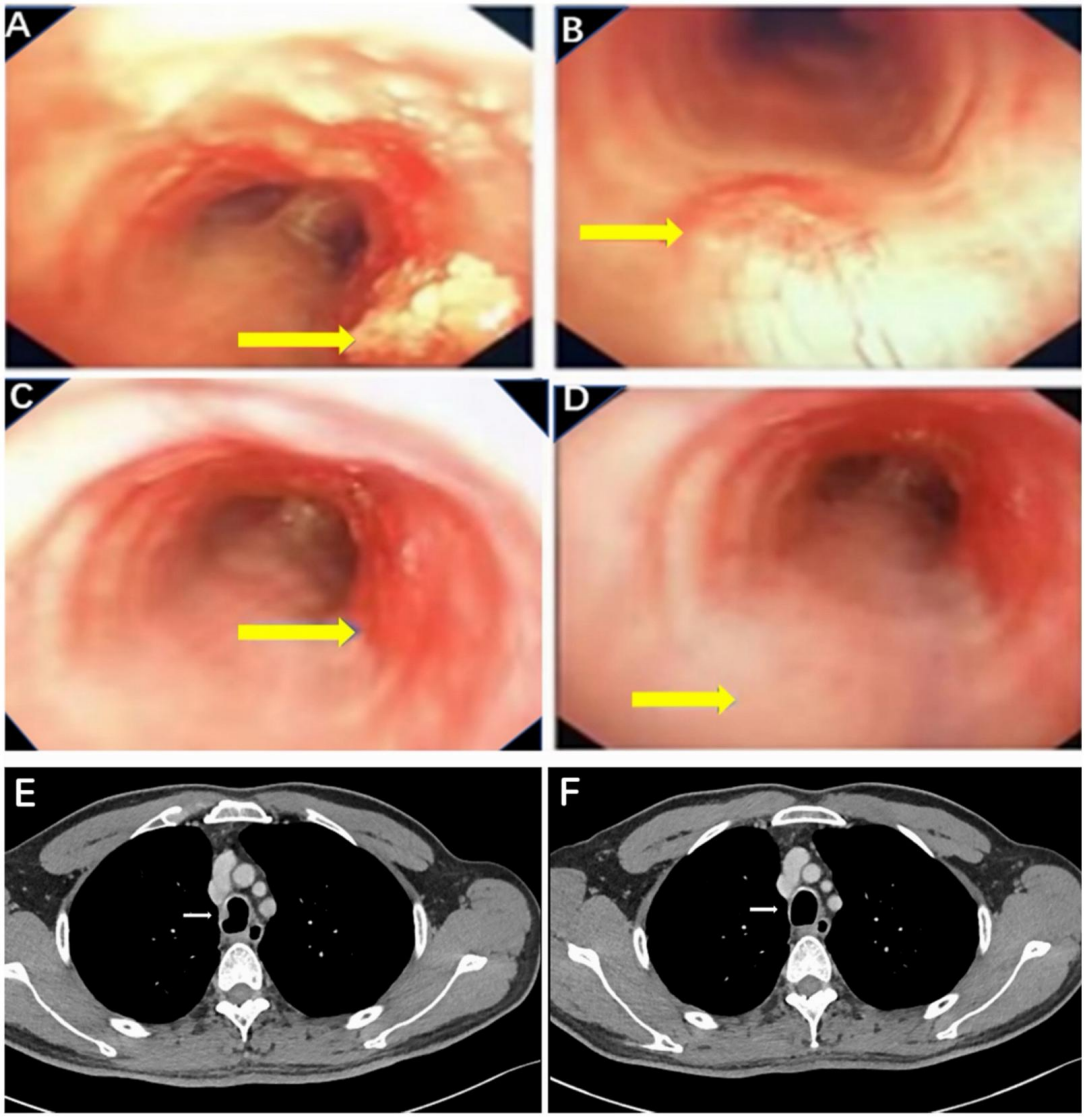
Treatment Response of Endotracheal Recurrent Lesions Evaluated by Contrasted CT and Bronchoscopy. (A) A polypoidal lesion on the right lateral mucosa lining of the trachea 5 cm above the carina (arrow). (B) An oval lesion on the membranous tracheal wall with a smooth surface (arrow). (C) Significant resolution of the polypoidal lesion after immunochemotherapy (arrow). (D) Complete resolution of the lesion on the membranous tracheal wall after immunochemotherapy (arrow). (E) The axial mediastinal window shows the recurrent lesion on the right lateral wall of the trachea before immunochemotherapy (arrow). (F) The axial mediastinal window of the same level shows a complete clinical response after immunochemotherapy (arrow).

After a 2-year follow-up, he presented with an irritative cough. Contrasted computed tomography (CT) and surveillance bronchoscopy revealed multifocal polypoidal lesions on the tracheal mucosa, 5 cm above the carina (**Figure 2A, B, and E**). Biopsy confirmed recurrent SCC (**Figure 1C and D**). Positron emission tomography/computed tomography (PET/CT) showed no distant metastases. Next-generation sequencing detected no actionable mutations. PD-L1 testing was precluded due to biopsy tissue exhaustion. Given the multifocal presentation and high risk of catastrophic complications following the prior sleeve lobectomy, local salvage interventions like reoperation or radiotherapy were prohibitive. Following multidisciplinary evaluation, the patient received first-line systemic immunochemotherapy: carboplatin (200 mg/m^2^, day 1-2), nab-paclitaxel (400 mg/m^2^, day 1), and nivolumab (200 mg, day 1) every 3 weeks for 4 cycles.

The patient tolerated the treatment well and experienced no adverse events related to therapy. Surveillance imaging and bronchoscopy demonstrated complete macroscopic resolution of the lesions and irritative cough (**Figure 2C, D, and F**). He subsequently transitioned to maintenance nivolumab (200 mg every 3 weeks). As of the latest evaluation (14 months post-induction), the patient maintains an ongoing complete clinical response with a durability of response (DOR) of 12 months, without delayed adverse events.

## DISCUSSION

Airway mucosal recurrence from primary SCC is atypical. However, surgical trauma from the prior sleeve lobectomy may have contributed to a compromised natural mucociliary defence. Combined with the “field cancerization” effect typical of SCC, exfoliated tumour cells from the central airway could have facilitated implantation onto the damaged tracheal mucosa. Therefore, one possible explanation is that these multifocal lesions represent aerogenous mucosal dissemination rather than haematogenous metastasis.

Currently, there are no standard treatment strategies for airway mucosal recurrence from primary lung cancer. In addition, local salvage interventions (eg, radiotherapy or reoperation) were prohibitive for this patient due to the multicentricity and high risk of catastrophic complications near the prior sleeve anastomosis; hence, systemic therapy was the optimal choice. Although PD-L1 testing was precluded by biopsy tissue exhaustion, combining a PD-1 inhibitor with platinum-based chemotherapy is a NCCN Category 1 first-line intervention for driver-negative SCC, regardless of PD-L1 expression.^2^ This combination leverages biological synergy: chemotherapy provides rapid tumour debulking to prevent impending airway obstruction, while maximizing the durable efficacy of the PD-1 inhibitor.^3^

The combination of immune checkpoint inhibitors with chemotherapy has been reported^2^^,^^3^ in the literature as first-line therapy for NSCLC and as an option for patients with tracheal metastasis. Besides, the Checkmate-816 trial^4^ demonstrated the feasibility and safety of nivolumab plus chemotherapy in stage IB-IIIA NSCLC, with a remarkable EFS of 31.6 months, especially in SCC. Bradley et al^5^ also reported a favourable response to immunotherapy for recurrent tracheal SCC. Our study supplements the current literature by illustrating a marked response to immunochemotherapy in a patient with airway mucosal recurrence of SCC. Treatment with this PD-1 inhibitor plus chemotherapy may represent a potential systemic treatment option in selected patients with airway mucosal recurrence. However, further clinical trials will be needed to evaluate the safety and efficacy of this treatment approach.

## Data Availability

The data underlying this article will be shared on reasonable request to the corresponding author.
